# The Relationship Between Cognitive Performance Using Tests Assessing a Range of Cognitive Domains and Future Dementia Diagnosis in a British Cohort: A Ten-Year Prospective Study

**DOI:** 10.3233/JAD-210030

**Published:** 2021-05-04

**Authors:** Shabina A. Hayat, Robert Luben, Kay-Tee Khaw, Carol Brayne

**Affiliations:** Department of Public Health and Primary Care, University of Cambridge, Cambridge, UK

**Keywords:** Cognition, dementia, epidemiology, risk

## Abstract

**Background:**

Exploring the domains of cognitive function which are most strongly associated with future dementia may help with understanding risk factors for, and the natural history of dementia.

**Objective:**

To examine the association of performance on a range of cognitive tests (both global and domain specific) with subsequent diagnosis of dementia through health services in a population of relatively healthy men and women and risk of future dementia.

**Methods:**

We examined the association between performance on different cognitive tests as well as a global score and future dementia risk ascertained through health record linkage in a cohort of 8,581 individuals (aged 48–92 years) between 2004–2019 with almost 15 years follow-up (average of 10 years) before and after adjustment for socio-demographic, lifestyle, and health characteristics.

**Results:**

Those with poor performance for global cognition (bottom 10%) were almost four times as likely to receive a dementia diagnosis from health services over the next 15 years than those who performed well HR = 3.51 (95% CI 2.61, 4.71 *p* < 0.001) after adjustment for socioeconomic, lifestyle, and biological factors and also prevalent disease. Poor cognition performance in multiple tests was associated with 10-fold increased risk compared to those not performing poorly in any test HR = 10.82 (95% CI 6.85, 17.10 *p* < 0.001).

**Conclusion:**

Deficits across multiple cognitive domains substantially increase risk of future dementia over and above neuropsychological test scores ten years prior to a clinical diagnosis. These findings may help further understanding of the natural history of dementia and how such measures could contribute to strengthening future models of dementia.

## INTRODUCTION

There has been an increased interest in the prevention or delay of the onset of dementia [1] and of the early identification of individuals who are at high risk [2]. For the purpose of primary prevention, we need a better understanding of the natural progression of the disease to allow targeting of specific risk factors to potentially modify early biological changes that may be an indication of future dementia [3]. Although early diagnosis of dementia is recommended in individuals displaying symptoms of the condition [4], current policies do not support screening or risk prediction at an individual level among apparently healthy individuals [3]. This is due to uncertainty of clinical outcomes and effective interventions [5], with insufficient evidence of benefits over the harms of screening for cognitive impairment and dementia [4, 6]. Arguments against screening highlight these uncertainties, including unnecessary investigation and treatment with no evidence of any benefit for the individual [7, 8].

Dementia for most has a long prodromal period, whereby individuals who go onto develop dementia, exhibit cognitive deficits many years before any symptoms or receiving a clinical diagnosis [9]. Though difficult to discriminate dementia in its early stages from normal cognitive ageing [10–12], there is evidence from large cohort studies that, in general, those who developed dementia after follow-up exhibited poorer cognitive performance compared to those who did not develop the disease [13], although differences have been insufficient to translate into accurate individual risk prediction [3]. The increased risks have been observed for tests both at a global and domain specific level [14, 15]. This includes episodic memory, executive functioning, verbal ability, visuospatial skill, attention, and processing speed [16, 17].

Dementia risk reduction has been identified one of the top research priorities in reducing the global burden of dementia [18]. Though not clinically applied, numerous dementia risk models have been developed and are widely used for research. These models have a number of uses, such as to examine factors that are associated with increased or reduce risk of dementia, or to classify individuals into different risk categories and in particular, identify those with high risk [19] in order to and identify target populations for intervention and prevention trials [3]. The accuracy of dementia prediction models have been reported as variable among different cohorts [19, 20].

Neuropsychological testing provides information on the nature and extent of cognitive deficits, and has been a core measure included in models of dementia in addition to age [19, 21]. Many studies have been limited either by small cognitive batteries assessing few cognitive domains or using tools such as the widely used Mini-Mental State Examination [22] (MMSE) or the Montreal Cognitive Assessment (MoCA), both known to be less sensitive to milder levels of cognitive dysfunction [23–25]. It is necessary to evaluate the utility of tests and to identify tests that are sensitive to early changes [26].

Variability across tasks also indicates neurological dysfunction [27]. Greater variability across different cognitive domains has been associated with poorer performance and dementia [28] and has been said to be a good predictor of cognitive impairment over and above the mean level of performance in individual cognitive tasks [29]. Furthermore, there is also evidence of more pervasive cognitive deficits across domains in earlier stages of decline, and not just memory alone [13, 30, 31]. Dementia is characterized by severe deficits across a range of fluid cognitive abilities, which worsens and affects more domains as the disease progresses [32]. However, it is still unclear, whether the cognitive performance is associated with dementia across all cognitive functions or whether it is specific to one or more key functions. Further research with a wider range of neuropsychological tests is needed [33, 34].

As well as the limitation of studies with cognitive tests that are sensitive and broad-ranging [35], there are fewer longitudinal studies [36] with sufficient follow-up time [37]. Investigating the utility of cognitive tests in individuals free of cognitive impairment or dementia at the time of testing, across a wider age range merits further investigation. Although large prospective cohort studies have shown an association between pre-clinical cognitive capability and increased risk of dementia [21], there is little in the current literature, on the level of impairment by cognitive domain, and in studies with follow-ups of more than 10 years [37]. The purpose of this study is to provide additional insight, that is has not been clear in previous studies, into the extent of impairment in natural history of dementia to further inform potential primary prevention strategies. A greater understanding of the role of specific risk factors associated with dementia is needed not only to understand the natural history of dementia, but also to better inform future risk prediction models when selecting components to be included [20].

We examine the utility of a range of cognitive tests (both global and domain specific), that are more sensitive to earlier changes of decline as potential key components, for risk of future dementia. We investigate whether early signs of impairment across a number of cognitive tasks influences the associations with incident dementia over and above cognitive performance on individual tasks, in a relatively healthy ageing prospective cohort study of mid to later life.

## METHODS

### Study participants and data collection

The European Prospective Investigation of Cancer (EPIC) is a European wide study of diet and health of which EPIC-Norfolk is one collaborating center. At the inception of the study (1993–1997), EPIC-Norfolk recruited 25,639 community-dwelling men and women (40–79 years old) from GP registers in and around the city of Norwich (Norfolk, United Kingdom). This involved the completion of a health and lifestyle questionnaire and a clinical examination [38]. The data presented here are from the third health examination (3HC) which was conducted between 2006 and 2011 with a preceding pilot phase between 2004 and 2006. Participants were aged 48–92 years, with no report of overt cognitive problems at the time of cognitive testing. The full assessment was a comprehensive 3-h examination which included tests assessing different domains of cognitive function. A detailed description of the cohort both at inception and at 3HC have been published [39, 40].

The study was approved by the Norfolk Local Research Ethics Committee (05/Q0101/191) and East Norfolk and Waveney NHS Research Governance Committee (2005EC07L). The study also has approval for follow-up through record linkage (REC Ref 98CN01). Informed consent was obtained from all individual participants included in the study. This study was conducted in compliance with the principles expressed in the Declaration of Helsinki and the Research Governance Framework for Health and Social Care.

### Assessment of cognition

The EPIC-Norfolk cognition battery at the 3HC phase consisted of seven tests, assessing performance across different cognitive domains. One of the tests, Visual Sensitivity Test (VST) for processing speed, had two outcome measures, thus giving a total of eight separate cognitive measures. These test have been described previously [40] and are summarized in Table 1.

**Table 1:** List of the individual cognitive tests used in the EPIC-Norfolk

Name of Test	Predominant ability measured by test	(Description of score)
1	A shortened version of the Extended Mental State Exam (SF-EMSE)	Global function (continuous score)
2	Hopkins Verbal Learning Test (HVLT)	Verbal episodic memory (continuous score)
3	Cambridge Neuropsychological Test Automated Battery Paired Associates Learning Test. First trial Memory Score (CANTAB-PAL FTMS)	Non-verbal episodic memory (continuous score)
4	PW Letter Cancellation Task (PW-Accuracy Score)	Attention (continuous score)
5	Event and Time Based Task (prospective memory)	Prospective memory (dichotomous outcome, success or fail)
6	Visual Sensitivity Test (VST)^*^ (1)VST-Simple	Simple and complex visual processing speed measured in milliseconds (continuous score).
7	(2)VST-Complex
8	Shortened version of the National Adult Reading Test^*^ (short-NART)	Reading ability and crystallised intelligence (continuous score)

^*^Higher score corresponds to poorer performance.

### Covariates

Education and social class were ascertained from health and lifestyle questionnaire administered at baseline (1993–1997). Objective measures of co-variates, such as cholesterol, systolic blood pressure, body mass index (BMI), waist hip ratio (WHR), forced expiratory volume in one second (FEV1), blood pressure, and plasma vitamin C were obtained from the clinical examination and physical activity, and prevalent disease were ascertained from self-reported health and lifestyle questionnaire administered at the time of the clinic appointment

### Dementia ascertainment and diagnostic codes

Almost complete follow-up for disease outcomes in EPIC-Norfolk has been established via linkage to routinely collected National Health Service (NHS) databases in England (Hospital Episode Statistics, HES) and mortality data for all participants using their unique NHS number and date of birth. The linked hospital records contain coded diagnostic information for all inpatient and day-case admissions [41]. To maximize dementia ascertainment, we also obtained national mental healthcare data.

Incident dementias were defined as those people free of dementia at the time of enrolment to the study, and at the time of cognitive testing (at 3HC) but identified with a dementia diagnosis through routine records subsequently. Participants were followed up from the date of consent at baseline until the first date of a dementia diagnosis, date of death or censoring with neither at 31 March 2019, allowing the timing of dementia onset to be accurately ascertained. The hospital and mortality and hospital data are coded using the International Classification of Diseases version 10 (ICD-10) which are almost exclusively diagnostic codes. Here, dementia from HES records, death certificate, or the mental health data was defined as any of the ICD-10 codes as listed in Supplementary Table 1. We used cases with a definite clinical diagnosis from any cause dementia. The sub types of dementia were not analyzed separately in this study.

### Analyses

Associations were examined using approximate percentile cut-offs rather than the continuous cognitive score. Poor performance was defined as obtaining a score less than a cut-off point corresponding to approximately the 10th percentile of the population distribution in each of the eight cognitive measures individually. We have described previously, the rationale for using percentile cut-off’s in this population, where the prevalence of cognitive impairment using accepted standard diagnostic criteria is low and the cognitive scores were not normally distributed [42, 43]. It was therefore necessary to establish operational criteria for cognitive dysfunction specific to this population. Participants were classified into two groups based on the cut-off scores for each of the tests. For prospective memory, poor performance was defined as those failing the task. A composite score (EPIC-COGComp) was also created from the eight individual cognitive measures. This composite represents general cognition underlying all the cognitive functions assessed. Participants were classified in two groups for the continuous composite score in the same way as the scores were for the individual tests as described above.

The risk of a ‘definite dementia’ diagnosis was estimated as a hazard ratio with 95% confidence interval (95% CI) for each of the cognitive tests in separate Cox proportional hazard regression models. Age (at time of cognitive testing), and multivariable-adjusted models were additionally constructed to estimate dementia risk. The models were as follows:

Model 1: Socio-demographic factors (age, per 5 years, sex, education, and social class).

Model 2: Socio-demographic and lifestyle (smoking, physical activity, and alcohol).

Model 3: Socioeconomic, lifestyle, and biological factors (cholesterol, systolic blood pressure, BMI, WHR, FEV, and plasma vitamin C), and prevalent disease.

The distribution of participants with poor cognitive performance across the eight cognitive measures were categorized based on the number of tests with a poor performance score as follows: A, 4–8 tests; B, 2-3 tests; C, 1 test; D, 0 tests (Reference category). Associations were examined with the number of tests included as a categorical variable in in the multivariate adjusted model (Model 3), and then additionally adjusted for each cognitive test.

As a supplementary analysis, we used multivariable regression analysis to examine the potential value of adding level of cognitive impairment to improve the accuracy of predictive modelling for dementia. We generated predicted probabilities from multiple logistic regression, which were then used to plot a receiver operating characteristic (ROC) curve to derive the area under the curve (AUC). The predictor variables included models examined by ROC were:

A: Age, sex and, education (basic model, as reported in previously [21]).

B: Multi-variable adjusted model (socioeconomic, lifestyle, biological factors, and prevalent disease or Model 3).

C: Variables as in Model 3, also adjusted for composite score (using the dichotomous variable of the composite).

D: Variables as in Model 3, further adjusted for ‘number of tests with poor performance’ (using the 4-level variable for number of tests, as described above).

### Missing data

Hazard ratios were examined by assigning participants with missing data to either the poor performance or to the reference category. Hazard ratios also examined for individuals with data on all eight cognitive tests and the specified covariates (N = 6,151) and compared to those with complete missing data of any of the eight cognitive measures as well as those not attending the health examination.

### Sensitivity analyses

A number of sensitivity analyses were conducted. Firstly, we grouped participants in approximate quartiles of cognition scores to explore whether the relationship of cognition and risk of dementia was a continuum or threshold, using a different (less stringent) grouping of the cognition scores. Secondly, we excluded participants who had died or were diagnosed with dementia in the first five years of follow-up to test for potential reverse causation bias.

## RESULTS

**Fig. 1:**
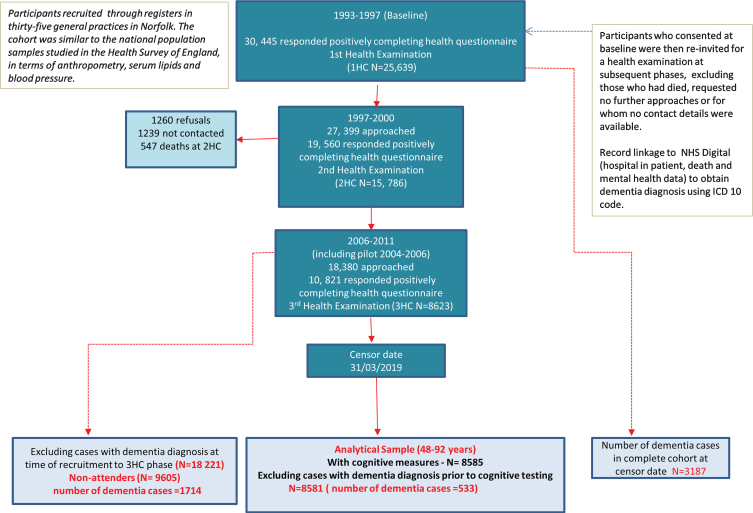
Selection of study participants in the EPIC-Norfolk third health check (including pilot phase 2004–2006) for all-cause ‘definite’ dementia, followed until 31 March 2019.

Of the 8,623 individuals who took part in the 3HC, 8,585 had cognitive measures with a total of 537 with a dementia diagnosis (based on the ICD codes in Supplementary Table 1) after a maximum of 14.8 years of follow up (mean of 9.6 and median of 9.8 years). Four of these participants were excluded from the analyses as they received their dementia diagnosis prior to their cognitive assessment resulting in a total of 533 dementia cases in the final analytical sample of 8,581 men and women who were aged 48–92 at the time of their cognitive assessment. Figure 1 summarizes the participation level at each phase of the study from baseline and the selection of the analytical sample. The total number of incident dementia in EPIC-Norfolk participants from the 25,639 individuals who attended the baseline health check at the censor date was 3,187.

**Fig. 2:**
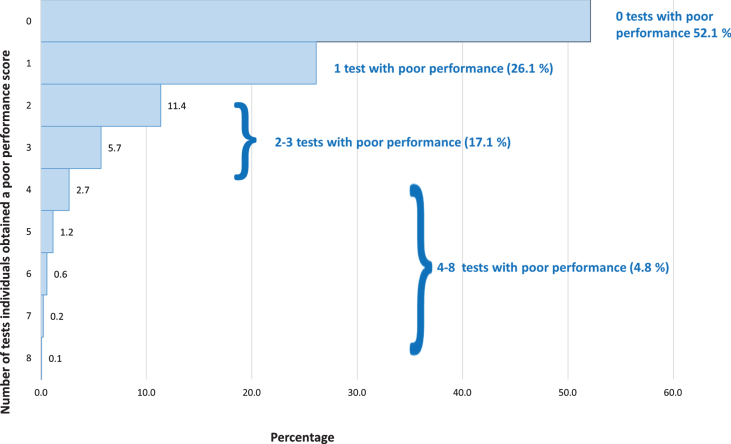
Distribution of poor performance across the eight measures in the EPIC-Norfolk cohort.

Table 2 shows the means and proportions of the variables included in this analysis by dementia status. As expected, there were differences between the two groups for almost all the variables examined. Those with dementia were more likely to be older, have no qualifications, be physically inactive and non-drinkers, less likely to be current smokers, and have higher blood pressure. They were also more likely to have reported suffering from stroke, hypertension, diabetes, and memory and hearing problems. Those with subsequent dementia scored lower on all the cognition tests at the 3HC, except the short-NART, which is a test of reading ability and crystallized knowledge. Of the 8,581 participants with cognitive data, 6,152 participants had data for all the cognitive tests with 2,391 having some of the test measures and 38 participants having none. In the healthier cohort, over half the participants did not exhibit poor performance in any of the eight cognitive measures, 26.1% exhibited poor performance in one test, 17.1% in 2-3 tests, and 4.8% in 4–8 tests (Fig. 2).

**Table 2:** Characteristics by dementia status of 8,581 participants with cognitive measures in the Third Health Check Phase of the European Prospective Investigation of Cancer in Norfolk (EPIC-Norfolk) study, 2006–2011 (including pilot data, 2004–2006). Participants followed up until 31 March 2019

	Definite dementia		No dementia		*p*
	N = 537		N = 8,048	
*Socio-demographic*
Mean (SD)
Age	76.3	(6.2)	68.2	(7.9)	<0.001
Sex, % women (n)	50.3	(266)	55.6	(4476)	0.02
Marital status, % married (n)	69.8	(353)	78.8	(6204)	<0.001
Education, % (n)
No qualifications	34.0	(180)	25.7	(2068)	<0.001
O/ A level standard	51.0	(270)	56.5	(4548)
Graduate level	14.9	(79)	17.8	(1434)
Social class, % (n)
Professional	7.8	(41)	8.9	(707)	0.5
Managerial	41.7	(220)	41.1	(3278)
Skilled non-manual	17.3	(91)	16.0	(1272)
Skilled manual	18.8	(99)	20.7	(1647)
Semi-skilled	12.7	(67)	11.1	(883)
^*^Retired	76.3	(5904)	95.8	(483)	<0.001
Non-skilled	1.7	(9)	2.3	(187)
*Lifestyle*
Physically inactive, % (n)	46.2	(237)	36.6	(2909)	<0.001
Smoking status, % (n)
Current	3.3	(17)	4.4	(353)	0.01
Former	52.6	(270)	45.5	(3620)
Never	44.1	(226)	50.0	(3976)
Alcohol intake, % (n)
0 units	36.4	(180)	29.4	(2281)	0.004
1–14 units/week	54.1	(268)	59.1	(4586)
>14 units per week	9.5	(47)	11.5	(896)
Take part in regular social activities	66.7	(171)	64.4	(2830)	0.3
*Biological/physiological*
Body mass index (Kgs/M^2^)	26.7	(4.2)	26.8	(4.3)	0.4
Waist hip ratio	0.91	(0.08)	0.89	(’(0.08)	0.003
Total cholesterol in mmol/L	5.1	(1.17)	5.4	(1.1)	<0.001
Systolic blood pressure, mmHg	138.5	(18.0)	136.0	(16.2)	0.001
Plasma vitamin C in umol/L	60.5	(22.4)	63.2	(22.3)	0.02
FEV (mL)	2.18	(0.7)	2.46	(0.7)	<0.001
*Prevalent disease*
Co-morbidity, self-report Yes% (n)
Heart attack	4.0	(21)	3.4	(270)	0.5
Hypertension	37.8	(200)	24.8	(2000)	<0.001
Stroke	4.5	(24)	2.0	(158)	<0.001
Cancer	10.2	(54)	9.3	(750)	0.5
Diabetes	6.8	(36)	2.8	(224)	<0.001
Depression	18.9	(100)	21.9	(1762)	0.1
COPD	9.3	(49)	8.1	(653)	0.4
Memory problems	7.4	(39)	1.9	(152)	<0.001
Hearing problems	41.2	(218)	31.0	(2498)	<0.001
Cognitive Test Score, Mean (SD)
SF-EMSE	29.8	(4.4)	32.8	(2.9)	<0.001
HVLT	20.0	(6.4)	25.4	(5.5)	<0.001
FTMS	12.7	(4.7)	15.8	(4.2)	<0.001
PW-Accuracy	9.7	(6.6)	13.4	(5.9)	<0.001
VST-Simple	722.0	(224.6)	660.2	(161.4)	<0.001
VST-Complex	2378.0	(500.0)	2185.9	(421.7)	<0.001
NART Error	17.4	(10.0)	17.2	(9.9)	0.7
Success frequency % (N)
Prospective memory	82.8	(6537)	57.5	(289)	<0.001

^*^From main occupation at time of cognitive testing P values by T test or Chi sq for proportion. HVLT, Hopkins Verbal Learning Test, ms, milliseconds; N, number; PAL-FTMS, Paired Associated Learning, First Trial Memory Score; Pros. Mem, Prospective memory; PW-Acc, PW-Accuracy, SD, standard deviation; SF-EMSE, Short Form Extended Mental State Exam; Sh-NART, Short National Adult Reading Test; VST, Visual Sensitivity Test.

Hazard ratios for dementia, adjusted for age (at the time of the invitation to the 3HC) per 5 years, sex, education, and social class for those who attended the health check and those who were invited but did not attend (N = 9,605 –as shown in Fig. 2) were examined. Using the group who had attended 3HC and had data on all 8 tests as reference, the dementia risk (adjusted for age, sex, education, and social class) were as follows: with data on 5–7 tests, HR = 1.12 (95% CI 0.93, 1.35 *p* = 0.2); 1–4 tests HR = 1.38 (95% CI 0.95, 2.00 *p* = 0.1); attended 3HC, but with no cognition data HR = 2.35 (95% CI 1.21, 4.57 *p* = 0.01), and for those who were invited for the 3HC but did not attend HR = 1.83 (95% CI 1.61, 2.08 p≤0.001). Data shown in Supplementary Table 2.

Table 3 shows the results of the Cox proportional hazards analysis for all the tests separately and for the composite score. For the age and sex adjusted models, there was an increased risk of dementia in those obtaining a poor performance score as compared to those who did not for all the cognitive tests other than the short-NART. Further adjustments for education and social class (Model 2) and then for co-variates, smoking, body mass index, physical activity, and comorbidities (Model 3) made little difference to the hazard ratios. The magnitude of the association varied slightly across tests, with the association with the composite as the strongest. Of the individual tests, the HVLT, a test for verbal episodic memory was comparable to the association observed for the composite.

**Table 3:** Association of cognitive performance (using the bottom 10th percentile as cut-off for poor performance) and dementia across the eight cognitive measures separately and a combined composite score as measured in the EPIC-Norfolk Cohort (2006–2010), including pilot data (2004–2006)

		Model 1				Model 2				Model 3			
Test, freq (N)	Frequency dementia % (N)*	Dementia (N)	HR	95% CI	p	Dementia (N)	HR	95% CI	p	Dementia (N)	HR	95% CI	p
SF-EMSE (8479)		**521**				**519**				**351**
Poor	18.4 (201)		3.09	(2.58, 3.70)	<0.001		3.20	(2.67, 3.87)	<0.001		3.16	(2.51, 3.98)	<0.001
Good	4.3 (320)		1.00				1.00				1.00
	p<0.001
HVLT (8135)		**485**				**484**				**333**
Poor	21.2 (175)		3.45	(2.84, 4.18)	<0.001		3.56	(2.93, 4.34)	<0.001		3.12	(2.44, 4.00)	<0.001
Good	4.2 (310)		1.00				1.00				1.00
	p<0.001
FTMS (7459)		**437**				**436**				**289**
Poor	15.4 (126)		2.06	(1.66, 2.55)	<0.001		2.05	(1.66, 2.55)	<0.001		2.11	(1.61, 2.78)	<0.001
Good	4.7 (311)		1.00				1.00				1.00
	p<0.001
PW-Acc (8406)		**510**				**508**				**343**
Poor	13.7 (133)		1.79	(1.47,2.19)	<0.001		1.82	(1.49, 2.23)	<0.001		1.78	(1.39, 2.28)	<0.001
Good	5.1 (377)		1.00				1.00				1.00
	p<0.001
VST-Simple (7169)		**413**				**412**				**289**
Poor	11.9 (85)		1.77	(1.39, 2.25)	<0.001		1.80	(1.41, 2.29)	<0.001		1.78	(1.33, 2.38)	<0.001
Good	5.1 (328)		1.00				1.00				1.00
	p<0.001
VST-Complex (7169)		**413**				**412**				**289**
Poor	14.8 (106)		2.15	(1.72, 2.69)	<0.001		2.17	(1.73, 2.72)	<0.001		2.18	(1.65, 2.86)	<0.001
Good	4.8 (307)		1.00				1.00				1.00
p<0.001
NART errors (8109)		**474**				**472**				**330**
Poor	6.5 (55)		1.10	(0.83, 1.46)	0.4		1.10	(0.83, 1.46)	0.5		1.07	(0.73, 1.56)	0.7
Good	5.8 (419)		1.00				1.00				1.00
	p = 0.5
Prospec. memory (8400)		**507**				**505**				**341**
Failure (1574)	13.7 (216)		2.37	(1.98, 2.84)	<0.001		2.37	(1.97, 2.84)	<0.001		2.36	(1.89, 2.95)	<0.001
Success (6826)	4.3 (291)		1.00				1.00				1.00
	p<0.001
Composite score (6151)		**334**				**333**				**230**
Poor	19.4 (124)		3.50	(2.75, 4.35)	<0.001		3.71	(2.93, 4.70)	<0.001		3.51	(2.61, 4.71)	<0.001
Good	3.8 (210)		1.00				1.00				1.00
	p<0.001

Model 1: Socio-demographic (Age, per 5 years, sex, education, and social class) factors. Model 2: Socio-demographic and lifestyle (smoking, physical activity, and alcohol) factors. Model 3: Socioeconomic, lifestyle, and biological (cholesterol, systolic blood pressure, BMI, WHR, FEV, and plasma vitamin C) factors and prevalent disease. HVLT, Hopkins Verbal Learning Test, ms, milliseconds; N, number; PAL-FTMS, Paired Associated Learning, First Trial Memory Score; Pros. Mem, Prospective memory; PW-Acc, PW-Accuracy; SD, standard deviation; SF-EMSE, Short Form Extended Mental State Exam; Sh-NART, Short National Adult Reading Test; VST, Visual Sensitivity Test.

In the sensitivity analysis, imputing missing into the poor performance or reference group, other than some slight attenuation made little difference to the hazard ratios (Supplementary Table 3). The analyses based on quartiles on cognitive performance showed no dose association in the quartile analyses, particularly at the top end of performance scores. (Supplementary Table 4). Therefore, using the more stringent cut-off in this cohort of healthier and higher functioning individuals is appropriate as presented in the main analysis. Repeating the multivariable analysis after excluding individuals who died or received a dementia diagnosis within five years of follow-up after cognitive testing (Number of dementia cases = 426), resulted in slight attenuation of the hazard ratios (Supplementary Table 5).

Associations based on the proportion with poor level of function across the cognitive abilities showed a steep linear increase in risk of dementia, in individuals with increasing numbers of abilities with a poor performance score (Table 4). Compared to those who did not have a poor performance score in any test, those with poor performance in one test had double the risk of dementia, poor performance in 2-3 tests had a four-fold increase, and those with poor performance in 4–8 tests, had over a ten-fold increased risk of dementia, HR = 10.82 (95% CI 6.85, 17.1 *p* = 0.001).

**Table 4:** Association between number of tests with a poor performance score and dementia, adjusting for all the co-variates (Model 3) and excluding those with dementia prior to attending the 3HC (N = 4,485)

Model 3
Number of tests where participants obtained a poor performance score	Frequency dementia (N)	HR	95% CI	p
	**230**
0 (N = 2365)		1.00
1 (N = 1184)		2.18	(1.45,3.27)	<0.001
2-3 (N = 745)		4.30	(2.90, 6.39)	<0.001
4–8 (N = 200)		10.82	(6.85, 17.1)	<0.001

Model 3: Socioeconomic, lifestyle, and biological factors and prevalent disease.

**Fig. 3:**
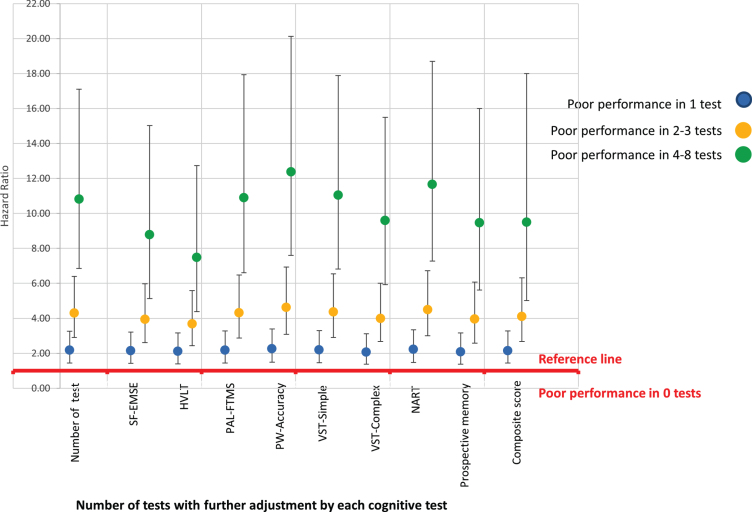
Diagrammatic representation of association of level of cognitive impairment and dementia controlling for each of the cognitive test.

Those with poor cognition in 4–8 tests showed more variability across domains than those with poor cognition in fewer domains. Controlling for each of the cognitive test as well as the composite score made little difference to the associations, as shown diagrammatically in Fig. 3 (with the data presented in Supplementary Table 6); however, adjusting for HVLT (episodic memory) did attenuate the association more than the other cognitive measures. Those who with poor performance in 4–8 tests were more likely to be older, have no qualifications, have higher reporting rate of heart attack, hypertension, stroke, diabetes, and memory and hearing problems. The mean score for all the cognition tests were substantially lower for this group, including a much higher NART error score. (Supplementary Table 7).

The supplementary analysis of the ROC curves as shown in Supplementary Figure 1 show the accuracy of the models presented in this study. The AUC successively improved from the basic model with addition of the covariates (Model 3), further with cognitive measure (shown here with the composite score); with the greatest predictive power of the final model which included ‘number of tests’. AUC values for these models for the individual tests (with the odds ratios from the multiple logistic regression used to generate the predicted probabilities) are presented in Supplementary Tables 8 and 9. These findings need to be examined in further studies

## DISCUSSION

We report on eight simple cognitive measures for which poor performance is associated with increased future risk of dementia in individuals with almost fifteen years of follow up. Impairment occurs in multiple domains several years prior to any clinical symptoms, and the more pervasive and greater the variability, the higher the risk of dementia. Our study presents additional evidence on the utility of a range of cognitive tests and the risk of dementia in this cohort of men and women in mid to later life who were free of dementia at the time of cognitive testing.

Impairment in multiple domains independently were associated with an increased risk of dementia over and above performance score of individual tests or a composite score. Poor cognition in four or more tests is associated with a ten-fold increased risk of developing dementia compared to those not performing poorly in any test. To our knowledge, no other study has examined associations with future incident dementia in detail, across a broad range of cognitive tests. Previous studies have either used a global composite or standardized score and not examined the relationship to include extent of impairment, nor a longer follow-up time as we have done here.

We have added to the current knowledge that extent of dysfunction, even at an earlier and milder level, has a substantial increased risk of future dementia. Adding cognition score to the multivariable adjusted model alone, showed an improvement in the accuracy of the model (AUC = 0.83 and 0.81, respectively), a finding that is comparable to other studies of risk prediction models [19, 21]. However, by incorporating the number of tests with impaired cognition, the model improved even further (AUC = 0.85). These findings provide further insight to cognitive predictors commonly included as components in risk prediction and could potentially inform future dementia prediction models. This is a novel and important finding and warrants further investigation. Although examining risk prediction is beyond the scope of this current study, the impact of adding level of impairment to accuracy of the model for a future risk prediction study should be examined in other studies.

There are a number of limitations to this study. The first is of healthy volunteer bias of those individuals attending the 3HC phase. However, our study included a wide range of individuals in terms of age, education, social class, and both men and women as in the general population [42]. Although EPIC-Norfolk was originally initiated as a general health study, the focus of the 3HC was on aspects of ageing, and so participants attending could have had concerns about their cognition. Those who did not attend this interview but had given permission to track medical records had 83% higher risk of dementia than those who attended and did all eight cognitive test HR = 1.83 (95% CI 1.61, 2.08 p≤0.001). While the absolute rates of dementia in the participants who undertook the cognitive function testing may not reflect those of a more general population, nevertheless there was still a wide range of ability in terms of cognitive performance.

The use of a self-report measure of many of the factors may also be criticized as prone to recall biases or not accurate as an objective measure. Conducting this study in this healthier population has the advantage of less confounding from co-morbidities. Although we adjusted for a wide range of factors, due to the nature of an observational study, we cannot exclude residual confounding. Given that this study is in relatively healthy older adults, we used a relatively stringent cut-off (even though the more stringent cut-off will still include cognitively poor but healthy individuals).

Using medical records allows a more complete follow-up, limiting attrition as a bias and is widely used method in epidemiological research [21]. The downside of this is the dependence on medical records, prone to inconsistencies across time, with changes in policy and practice raising concerns over the accuracy and changing completeness of dementia recording [44, 45]. Also, medical records are known to underestimate the number of individuals with dementia, as not all individuals receive a formal diagnosis. Nevertheless, even though less sensitive, medical record diagnosis is likely to be highly specific such that when an individual has a diagnosis or death certification with dementia recorded this is highly likely to be accurate [45].

Although we found minimal change in associations when omitting in sensitivity analyses those who had died or had a dementia diagnosis within 5 years of cognitive testing, we cannot rule out reverse causation as dementia has such a long preclinical phase [46]. Further follow-up time is needed. However, determining temporality for dementia will always be challenging. Also, having a wide age range at baseline, we were able to observe the different influence of factors and how their relationship varied with dementia as midlife or later life exposures.

Substantial cognitive changes occur with healthy ageing. Previous studies have shown that there are milder or pre-symptomatic stages of dementia, not limited to memory [30, 31]. We have confirmed these findings in this larger cohort with a wider age range. It is quite possible to misclassify milder symptoms or asymptomatic without memory concerns as normal cognitive ageing. Our findings show the magnitude of the association between a wide range of cognitive tests from the same cohort, with some tests more strongly associated than others. In particular, we show tests of processing speed are not as strongly related as the other tests. These points are important to consider in future work.

Variation in methodology, characteristics of the population, the timing and nature of cognitive test will all influence the association. It is essential to disentangle confounding to get a better understanding of the causal pathways and those at greatest risk. In our study, the future relative risk was ten-fold greater in those with pervasive impairment. Other cohort studies examining dementia risk should consider impairment across multiple domains to examine the association within their settings.

It is important to highlight, that even with a ten-fold increased risk of dementia in those with more pervasive impairments, there is insufficient evidence to advocate screening or use of predictive modelling for diagnostic use for dementia. There is still much to understand in terms of to provide further insight into the different domains cognitive function commonly used for assessment as components in dementia risk modelling. These findings may be used to develop future prediction models to identify earliest phases of impairment across cognitive domains, to inform the design of trials of preventive or modifying interventions [5] and identify target populations greatest at risk who can then also be included in such trials.

## Supplementary Material

Supplementary Material

## Data Availability

EPIC-Norfolk researchers will make the dataset available under a Data Transfer Agreement to any bona fide researcher who wishes to obtain the dataset in order to undertake a replication analysis. Although the dataset is anonymized, the breadth of the data included and the multiplicity of variables that are included in this analysis file as primary variables or confounding factors, means that provision of the dataset to other researchers without a Data Transfer Agreement would constitute a risk. The contact for data request is: EPIC-Norfolk@mrc-epid.cam.ac.uk.
