# Supplementary material for: The Relationship Between Cognitive Performance Using Tests Assessing a Range of Cognitive Domains and Future Dementia Diagnosis in a British Cohort: A Ten-Year Prospective Study
Source: J Alzheimers Dis. 2021 May 4;81(1):123–35. doi: 10.3233/JAD-210030 (PMC8203214; doi:10.3233/JAD-210030)
Supplement: Supplementary Material [file jad-81-jad210030-s001.pdf]

Supplementary Material

The Relationship Between Cognitive Performance Using Tests Assessing a Range of Cognitive Domains and Future Dementia Diagnosis in a British Cohort: A Ten-Year Prospective Study

Supplementary Table 1. ICD 10 codes used in EPIC-Norfolk to identify 'Definite' dementia cases (N=3173 in complete cohort from baseline of 30,445)

ICD Code	ICD Description
**F00**<br>**G30**	Dementia in Alzheimer's disease<br>Alzheimer's disease
**F00.0**<br>**G30.0**	Dementia in Alzheimer's disease with early onset
**F00.1**<br>**G30.1**	Dementia in Alzheimer disease with late onset
**F00.2**	Dementia in Alzheimer disease, atypical or mixed type
**G30.8**	Other Alzheimer's disease
**F00.9**<br>**G30.9**	Dementia in Alzheimer's disease, unspecified
**F01**	Vascular dementia
**F01.0**	Vascular dementia of acute onset
**F01.1**	Multi-infarct dementia
**F01.2**	Subcortical vascular dementia
**F01.8**	Other vascular dementia
**F01.9**	Vascular dementia, unspecified
**F02**	Dementia in other diseases classified elsewhere
**F02.0**	Dementia in Pick's disease
**F02.1**	Dementia in Creutzfeldt-Jakob disease
**F02.2**	Dementia in Huntington's disease
**F02.3**	Dementia in Parkinson's disease
**F02.8**	Dementia in other specified diseases classified elsewhere
**G31.0**	Frontotemporal dementia
**G31.8**	Other specified degenerative diseases of nervous system. Grey-matter degeneration [Alpers]<br>Lewy body(ies)(dementia)(disease).Subacute necrotizing encephalopathy [Leigh]
**F03**	Unspecified dementia
**F05.1**	Delirium superimposed on dementia
**F10.7**	Residual and late-onset psychotic disorder: Includes Alcoholic dementia NOS<br>Chronic alcoholic brain syndrome<br>Dementia and other milder forms of persisting impairment of cognitive functions

ICD-10, International Classification of Diseases -10th revision

Supplementary Table 2. Hazard ratios for dementia risk for those who attended the health examination and completed some cognitive tests and for those who were invited but did not attend, adjusted for age (at the time of the invitation to the 3HC) per 5 years, sex, education, and social class

**Attended 3HC**							**Invited but did not attend 3HC**	
8 Tests	5-7 Tests		1-4 tests		0 Tests			
HR	HR	95% CI	HR	95% CI	HR	95% CI	HR	95% CI
1.00 (Ref)	1.12	(0.93, 1.35)	1.38	(0.95, 2.00)	2.35	(1.21, 4.57)	1.83	(1.61, 2.08)
	p=0.2		p=0.1		p=0.01		p≤0.001	

Supplementary Table 3. Sensitivity analysis to examine the impact of missing data on association of poor performance with dementia for each cognition score separately and the composite score

	No Missing Values (Model 3)			'Missings' assigned to 'poor performance' group (Model 3) Dementia N=362			'Missings' assigned to 'reference' group (Model 3), Dementia N=362		
**Test**	**HR**	**95% CI**	**p**	**HR**	**95% CI**	**p**	**HR**	**95% CI**	**p**
**SF-EMSE**									
Poor	3.16	(2.51, 3.98)	<0.001	3.08	(2.46, 3.85)	<0.001	3.08	(2.45, 3.86)	<0.001
Good	1.00			1.00			1.00		
**HVLT**									
Poor	3.12	(2.44, 4.00)	<0.001	2.41	(1.92, 3.03)	<0.001	2.92	(2.30, 3.71)	<0.001
Good	1.00			1.00			1.00		
**FTMS**									
Poor	2.11	(1.61, 2.78)	<0.001	1.87	(1.50, 2.34)	<0.001	1.85	(1.43, 2.39)	<0.001
Good	1.00			1.00			1.00		
**PW-Acc**									
Poor	1.78	(1.39, 2.28)	<0.001	1.83	(1.45, 2.31)	<0.001	1.72	(1.34, 2.20)	<0.001
Good	1.00			1.00			1.00		
**VST- Simple**									
Poor	1.78	(1.33, 2.38)	<0.001	1.37	(1.10, 1.71)	<0.001	1.67	(1.26,2.21)	<0.001
Good	1.00			1.00			1.00		
**VST-complex**									
Poor	2.18	(1.65, 2.86)	<0.001	1.58	(1.28, 1.97)		2.05	(1.58, 2.67)	<0.001
Good	1.00			1.00			1.00		
**NART Errors**									
Poor	1.07	(0.73, 1.56)	0.7	1.08	(0.82, 1.43)	0.6	1.00	(0.69, 1.45)	0.9
Good	1.00			1.00			1.00		
**Prospec. Memory**									
Failure	2.36	(1.89, 2.95)	<0.001	2.38	(1.92, 2.95)	<0.001	2.22	(1.79, 2.76)	<0.001
Success	1.00			1.00			1.00		
**Composite score**									
Poor	3.51	(2.61, 4.71)	<0.001	2.58	(2.04, 3.26)	<0.001	2.95	(2.33,3.73)	<0.001
Good	1.00			1.00					

*Using Model 3 from the main analysis (adjusted for Socioeconomic, lifestyle and biological factors and prevalent disease). Hazard ratio for risk of dementia shown for (1) those with no missing values (2) missing values included as poor performers (3) missing values included in the reference (good) performers.

HVLT, Hopkins Verbal Learning Test, ms, milliseconds; N, number; PAL-FTMS, Paired Associated Learning, First Trial Memory Score; Prospec. Mem, Prospective memory; PW-Acc, PW-Accuracy, SD, Standard deviation; SF-EMSE, Short Form Extended Mental State Exam; Sh-NART, Short National Adult Reading Test; VST, Visual Sensitivity Test

Supplementary Table 4. Association of cognitive test score (by approximate quartile) and dementia across the eight cognitive measures separately and a combined composite score as measured in the EPIC-Norfolk 3 Cohort (2006-2010), including pilot data (2004-2006) and followed up over average of 9.6 years, after adjustment for covariates.

		**Model 1**				**Model 2**				**Model 3**			
**Test, Freq (N)**	**Frequency Dementia % (N)***	**Freq Dementia (N)**	**HR**	**95% CI**	**p**	**Freq Dementia (N)**	**HR**	**95% CI**	**p**	**Freq Dementia (N)**	**HR**	**95% CI**	**p**
**SF-EMSE (8479)**		**521**				**519**				**351**			
Q1 (2302)	13.2 (304)		3.57	(2.21, 5.78)	<0.001		3.82	(2.35, 6.20)	<0.001		4.18	(2.30, 7.61)	<0.001
Q2 (2276)	5.5 (126)		1.77	(1.08, 2.92)	0.03		1.84	(1.12, 3.02)	0.02		2.00	(1.09, 3.69)	0.03
Q3 (2897)	2.5 (73)		0.99	(0.56, 1.66)	0.9		1.01	(0.61, 1.70)	0.9		0.99	(0.52, 1.86)	1.00
Q4 (1004)	1.8 (18)		1.00				1.00				1.00		
	p<0.001												
**HVLT (8135)**		**485**				**484**				**333**			
Q1 (2034)	14.0 (285)		4.33	(3.02, 6.21)	<0.001		4.63	(3.21, 6.68)	<0.001		4.14	(2.65 6.47)	<0.001
Q2 (2514)	4.3 (109)		1.70	(1.16, 2.49)	0.001		1.75	(1.19, 2.56)	0.004		1.74	(1.10, 2.75)	0.02
Q3 (1640)	3.4 (55)		1.46	(0.96, 2.22)	0.08		1.47	(0.96, 2.24)	0.07		1.52	(0.92, 2.49)	0.1
Q4 (1947)	1.8 (36)		1.00				1.00				1.00		
	p<0.001												
**PAL-FTMS (7459)**		**437**				**436**				**289**			
Q1 (2073)	10.7 (221)		3.03	(1.92, 4.77)	<0.001		3.05	(1.93, 4.82)	<0.001		4.14	(2.22,7.75)	<0.001
Q2 (2093)	5.8 (121)		2.12	(1.33, 3.39)	0.002		2.13	(1.34, 3.40)	0.001		2.83	(1.50, 5.34)	0.001
Q3 (2012)	3.7 (74)		1.59	(0.98, 2.59)	0.06		1.62	(0.99, 2.63)	0.05		2.31	(1.20, 4.44)	0.04
Q4 (1281)	1.6 (21)		1.00				1.00				1.00		
	p<0.001												
**PW-Acc (8406)**		**510**				**508**				**343**			
Q1 (2103)	11.5 (241)		2.42	(1.77, 3.32)	<0.001		2.43	(1.77, 3.34)	<0.001		2.75	(1.83, 4.15)	<0.001
Q2 (2229)	6.5 (144)		1.54	(1.11, 2.14)	0.01		1.55	(1.12, 2.16)	0.01		1.81	(1.18, 2.75)	0.01
Q3 (2153)	3.5 (76)		1.04	(0.72, 1.49)	0.8		1.03	(0.72, 1.48)	0.9		1.28	(0.81, 2.01)	0.3
Q4 (1921)	2.6 (49)		1.00				1.00				1.00		
	p<0.001												

Supplementary Table 4 (continued)

		**Model 1**				**Model 2**				**Model 3**			
**Test, Freq (N)**	**Frequency Dementia % (N)***	**Freq Dementia (N)**	**HR**	**95% CI**	**p**	**Freq Dementia (N)**	**HR**	**95% CI**	**p**	**Freq Dementia (N)**	**HR**	**95% CI**	**p**
**VST- Simple (7169)**		**413**				**412**				**289**			
Q1 (1789)	8.9 (159)		1.47	(1.10, 1.96)	0.01		1.45	(1.09, 1.94)	0.01		1.33	(0.94, 1.87)	0.1
Q2 (1795)	5.5 (99)		1.04	(0.76, 1.41)	0.8		1.02	(0.75, 1.40)	0.9		0.94	(0.65, 1.37)	0.8
Q3 (1794)	4.8 (87)		1.03	(0.75, 1.42)	0.9		1.05	(0.77, 1.45)	0.8		1.05	(0.72, 1.53)	0.8
Q4 (1791)	3.8 (68)		1.00				1.00				1.00		
	p<0.001												
**VST-complex (7169)**		**413**				**412**				**289**			
Q1 (1791)	9.9 (177)		1.59	(1.20, 2.11)	0.01		1.61	(1.21, 2.13)	0.001		1.41	(1.01, 1.98)	0.04
Q2 (1793)	5.1 (92)		1.11	(0.81, 1.52)	0.5		1.11	(0.81, 1.52)	0.5		1.03	(0.71, 1.48)	0.9
Q3 (1793)	4.2 (75)		1.01	(0.73, 1.40)	1.0		1.01	(0.73, 1.40)	1.0		0.91	(0.62, 1.35)	0.6
Q4 (1792)	3.9 (69)		1.00				1.00				1.00		
	p<0.001												
**NART Errors (8109)**		**474**				**472**				**330**			
Q1 (1835)	6.2 (114)		0.93	(0.72, 1.19)	0.6		0.94	(0.70, 1.25)	0.7		0.85	(0.59, 1.21)	0.4
Q2 (2218)	5.9 (130)		0.89	(0.70, 1.14)	0.4		0.91	(0.70, 1.18)	0.5		0.86	(0.63, 1.18)	0.4
Q3 (2003)	5.2 (104)		0.80	(0.62, 1.04)	0.1		0.80	(0.62, 1.04)	0.1		0.83	(0.61, 1.13)	0.3
Q4 (2053)	6.1 (126)		1.00				1.00				1.00		
Composite score (6151)	p=0.5												
**Composite score (6151)**	**334**				**333**				**230**				
Q1 (1845)	10.8 (200)		3.82	(2.41, 6.06)	<0.001		4.16	(2.60, 6.66)	<0.001		4.87	(2.68, 8.85)	<0.001
Q2 (1368)	5.2 (71)		2.17	(1.33, 3.55)	0.002		2.22	(1.35, 3.64)	0.002		2.58	(1.38, 4.82)	0.003
Q3 (1401)	3.0 (42)		1.59	(0.94, 2.69)	0.08		1.62	(0.96, 2.75)	0.07		1.97	(1.02, 3.78)	0.04
Q4 (1537)	1.4 (21)		1.00				1.00				1.00		
	p<0.001												
**Prospec. Memory (8400)**		**507**				**505**				**341**			
Failure (1574)	13.7 (216)		2.37	(1.98, 2.84)	<0.001		2.37	(1.97, 2.84)	<0.001		2.36	(1.89, 2.95)	<0.001
Success (6826)	4.3 (291)		1.00				1.00				1.00		
	p<0.001												

Supplementary Table 5. Sensitivity analysis showing association with dementia after excluding individuals who died or were diagnosed with dementia within 5 years of cognitive testing (N=426 dementia cases).

		**Excluding death and dementia cases within 5 years***		
**Test, Freq (N)**	**Dementia (N)**	**HR**	**95% CI**	**p**
**SF-EMSE (7760)**	**288**			
Poor		2.63	(2.02, 3.41)	<0.001
Good		1.00		
**HVLT (7482)**	**277**			
Poor		2.8	(2.14, 3.72)	<0.001
Good		1.00		
**FTMS (6856)**	**240**			
Poor		1.80	(1.32, 2.46)	<0.001
Good		1.00		
**PW-Acc (7707)**	**283**			
Poor		1.54	(1.16, 2.05)	0.002
Good		1.00		
**VST- Simple (6594)**	**236**			
Poor		1.66	(1.18, 2.31)	0.003
Good		1.00		
**VST-complex (6594)**	**238**			
Poor		1.61	(1.16, 2.24)	0.01
Good		1.00		
**NART Errors (7074)**	**273**			
Poor		1.03	(0.68, 1.57)	0.9
Good		1.00		
**Prospec. Memory (7696)**	**283**			
Failure (1574)		2.25	(1.76, 2.87)	<0.001
Success (6826)		1.00		
**Composite score (5673)**	**189**			
Poor		2.94	(2.11, 4.11)	<0.001
Good		1.00		

Supplementary Table 6. Association of the number of cognitive tests and dementia with adjustment for co-variates (Model 3) and then additional adjustment for individual cognitive tests and composite score

		Model 3 (with no adjustment for any cog measure)			Model 3 and further adjusted for SF-EMSE			Model 3 and further adjusted for HVLT			Model 3 and further adjusted for FTMS			Model 3 and further adjusted for PW_Acc		
**Number of tests where participants obtained a poor performance score**	**Freq Dementia (N)**	**HR**	**95% CI**	**p**	**HR**	**95% CI**	**p**	**HR**	**95% CI**	**p**	**HR**	**95% CI**	**p**	**HR**	**95% CI**	**p**
	**321**															
0 (N=3138)		1.00			1.00						1.00			1.00		
1 (N=1567)		2.06	(1.47, 2.89)	<0.001	2.01	(1.43, 2.82)	<0.001	1.96	(1.40, 2.76)	<0.001	2.07	(1.47, 2.91)	<0.001	2.12	(1.51, 2.98)	<0.001
2-3 (N=1011)		3.69	(2.65, 5.14)	<0.001	3.34	(2.36, 4.73)	<0.001	2.98	(2.09, 4.23)	<0.001	3.76	(2.68, 5.29)	<0.001	4.00	(2.85, 5.62)	<0.001
4-8 (N=279)		9.15	(6.27, 13.37)	<0.001	7.20	(4.59, 11.30)	<0.001	5.54	(3.54, 8.68)	<0.001	9.52	(6.28, 14.43)	<0.001	10.44	(6.90, 15.58)	<0.001
	**321**															
0 (N=3138)		1.00			1.00			1.00			1.00			1.00		
1 (N=1567)		2.08	(1.48, 2.92)	<0.001	1.97	(1.40, 2.78)	<0.001	2.10	(1.50, 2.95)	<0.001	1.96	(1.38, 2.77)	<0.001	2.06	(1.47, 2.89)	<0.001
2-3 (N=1011)		3.77	(2.68, 5.29)	<0.001	3.50	(2.50, 4.91)	<0.001	3.88	(2.78, 5.42)	<0.001	3.39	(2.37, 4.85)	<0.001	3.23	(2.24, 4.67)	<0.001
4-8 (N=279)		9.48	(6.35, 14.15)	<0.001	8.36	(5.62, 12.44)	<0.001	10.11	(6.81, 15.01)	<0.001	7.95	(5.12, 12.34)	<0.001	6.45	(3.78, 11.02)	<0.001

Supplementary Table 7. Comparison of characteristics of individuals in the poor performance group across the eight cognitive measure

	0 tests	1 test	2-3 tests	4-8 tests	p
Socio-demographic					
**Mean (SD)**					
**Age**	65.9 (7.1)	68.8 (7.5)	71.4 (7.6)	74.5 (7.1)	<0.001
**Sex, % women (n)**	58.9 (1888)	54.7 (879)	47.1 (495)	46.4 (134)	<0.001
**Education as reported at baseline % (n)**					
No qualifications	15.2 (489)	28.1 (451)	40.6 (426)	52.8 (152)	
O/A level Standard	60.7 (1947)	56.4 (906)	51.2 (537)	43.8 (126)	<0.001
Graduate Level	24.0 (771)	15.5 (249)	8.2 (86)	3.5 (10)	
Prevalent disease					
**Co-morbidity, self report Yes% (n)**					
Heart attack	2.4 (77)	3.1 (49)	4.4 (46)	5.5 (16)	0.001
Hypertension	24.2 (777)	23.0 (369)	28.5 (299)	29.1 (84)	0.003
Stroke	1.3 (41)	1.8 (29)	2.4 (25)	7.3 (21)	<0.001
Cancer	8.7 (278)	8.9 (143)	11.0 (115)	8.7 (25)	0.2
Diabetes	2.5 (81)	2.7 (44)	3.4 (36)	6.2 (18)	0.003
Depression	22.6 (725)	21.9 (352)	18.9 (198)	22.1 (64)	0.1
COPD	8.5 (273)	8.3 (134)	6.7 (70)	5.9 (17)	0.1
self-report mem. Problem	0.9 (28)	1.6 (25)	3.7 (39)	12.1 (35)	<0.001
Hearing Problems	28.9 (928)	32.3 (518)	33.2 (349)	38.4 (111)	0.001
**Cognitive Test Score, Mean (SD)**					
SF-EMSE	34.0 (1.7)	32.9 (2.2)	30.9 (2.8)	27.6 (3.2)	<0.001
HVLT	27.4 (4.2)	25.1 (4.9)	21.5 (5.4)	16.3 (5.3)	<0.001
PAL- FTMS	17.8 (3.2)	15.5 (3.9)	13.3 (4.3)	9.9 (4.4)	<0.001
PW-Accuracy	15.6 (4.7)	12.8 (5.7)	9.4 (6.3)	5.8 (6.1)	<0.001
VST-simple, reaction time	615.1 (66.3)	670.87 (141.8)	732.6 (196.3)	844.5 (294.2)	<0.001
VST-Complex, reaction time	2082.9 (269.8)	2223.9 (377.3)	2338.5 (488.1)	2600.0 (691.2)	<0.001
NART Error	13.8 (7.4)	18.1 (9.8)	23.1 (10.6)	27.9 (10.9)	<0.001
**Success Frequency % (N)**					
Prospective memory	100 (3207)	72.5 (1165)	56.4 (592)	23.5 (68)	<0.001

Supplementary Figure 1. ROC curves for the prediction of incident dementia according to (1) age, sex, and education only (2) Multivariable Model (Model 3) socioeconomic, lifestyle, and biological factors and prevalent disease) Model 3+ cognitive test performance (composite score) (4) Model 3 + number of tests with a poor performance score cognitive test performance. Sample sizes are (left-hand graph) 81,823 including 78 dementia cases; (right-hand graph) 145,068 participants

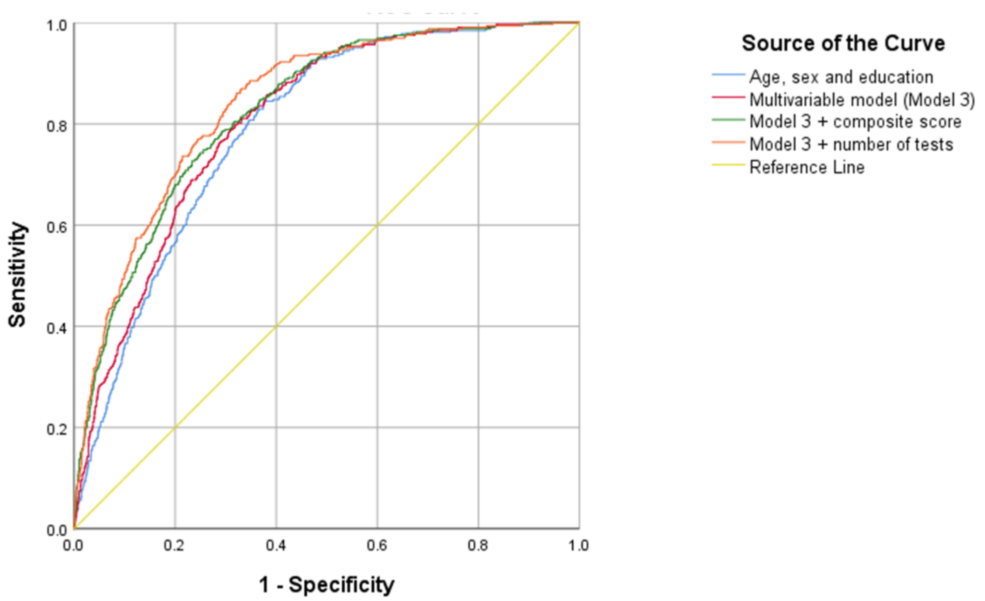

	AUC	95% CI	P-value
Age, sex, education	0.79	(0.77, 0.82)	<0.001
Model 3	0.81	(0.78, 0.83)	<0.001
Model 3+ Composite score	0.83	(0.81, 0.86)	<0.001
Model 3+ number of tests	0.85	(0.82, 0.87)	<0.001

Supplementary Table 8. Odds ratios (with 95%CI) of definite dementia diagnosis based on risk factors included (1) age, sex and education and (2) the fully adjusted and the multi-variate adjusted model (Model 3) as used in the cox regression as used in the main analysis. Also presenting AUC values as created from the predicted probabilities from multiple logistic regression.

**Risk factor**	**HR**	**95% CI**	**p**	**AUC**	**95%CI**	**p**
**Minimally adjusted model N=8579**				0.79	(0.77, 0.80)	<0.001
Age per 5 years	1.92	(1.81, 2.05)	<0.001			
Sex (Men)	1.07	(0.89, 1.29)	0.5			
Education (No Qualifications)	1.11	(0.91, 1.35)	0.3			
**Model 3 (Multi-variate model) N=8382**				0.80	(0.78, 0.81)	<0.001
Age per 5 years	1.94	(1.80, 2.08)	<0.001			
Sex (Men)	1.04	(0.86, 1.27)	0.7			
Education (No Qualifications)	1.12	(0.91, 1.38)	0.3			
Social class (Manual)	1.04	(0.84, 1.28)	0.7			
Smoking (current)	1.37	(0.83, 2.27)	0.2			
Physical activity (Inactive)	0.83	(0.69, 1.01)	0.07			
**Co-morbidity, self report Yes% (n)**						
Heart attack	0.53	(0.32, 0.88)	0.01			
Hypertension	1.35	(1.11, 1.64)	0.003			
Stroke	1.41	(0.85, 2.33)	0.2			
Cancer	0.80	(0.58, 1.09)	0.2			
Diabetes	1.85	(1.24, 2.77)	0.002			
Depression	1.16	(0.91, 1.47)	0.2			
COPD	1.11	(0.80, 1.53)	0.6			
Memory problems	2.25	(1.52, 3.34)	<0.001			
Hearing Problems	1.04	(0.86, 1.27)	0.7			

Multiple logistic regression was used to generate predicted probabilities for three models (1) adjusted for age, sex and education; (2) the multivariate model with all co-variates (Model 3) excluding the cognitive test; and (3) Model 3 plus cognitive performance for each of the tests and the composite score using both quartile and tenth percentile cut-offs (See Supplementary Table 6).

Supplementary Table 9. Odds ratios (with 95%CI) of dementia from the multiple logistic regression of the multi-variate model (Model 3) with additional adjustment for the eight cognitive measure, composite score and number of tests. Both quartile and tenth percentile cut-offs shown with corresponding AUC values for each model.

Test, Freq (N)	HR	95% CI	p	AUC	95%CI	p
**SF-EMSE (8285)**						
Quartiles				0.82	(0.80, 0.84)	<0.001
Q1 (2231)	3.97	(2.41, 6.55)	<0.001			
Q2 (2234)	1.93	(1.15, 3.22)	0.01			
Q3 (2835)	1.05	(0.62, 1.78)	0.9			
Q4 (985)	1.00					
10th PCTILE				0.82	(0.80, 0.83)	<0.001
Poor	3.14	(2.53, 3.89)	<0.001			
Good	1.00					
**HVLT (7951)**						
Quartiles				0.82	(0.80, 0.84)	<0.001
Q1 (1977)	3.92	(2.68, 5.73)	<0.001			
Q2 (2459)	1.66	(1.12, 2.46)	0.001			
Q3 (1612)	1.43	(0.93, 2.22)	0.1			
Q4 (1903)	1.00					
10th PCTILE				0.82	(0.80, 0.84)	<0.001
Poor	3.49	(2.78, 4.39)	<0.001			
Good	1.00					
**FTMS (7281)**						
Quartiles				0.81	(0.79, 0.83)	<0.001
Q1 (2005)	3.07	(1.91, 4.92)	<0.001			
Q2 (2053)	2.23	(1.38, 3.62)	0.001			
Q3 (1962)	1.81	(1.10, 2.98)	0.02			
Q4 (1261)	1.00					
10th PCTILE				0.81	(0.79, 0.83)	<0.001
Poor	1.98	(1.54, 2.54)	<0.001			
Good	1.00					
**PW-Acc (8215)**						
Quartiles				0.81	(0.79, 0.82)	<0.001
Q1 (2040)	2.31	(1.65, 3.25)	<0.001			
Q2 (2174)	1.55	(1.10, 2.21)	0.01			
Q3 (2120)	1.04	(0.71, 1.52)	0.8			
Q4 (1881)	1.00					
10th PCTILE				0.81	(0.79, 0.82)	<0.001
Poor	1.79	(1.42, 2.26)	<0.001			
Good	1.00					

Supplementary Table 9 (continued)

	Based on Model 3					
**Test, Freq (N)**	**HR**	**95% CI**	**p**	**AUC**	**95%CI**	**p**
**VST- Simple (6997)**						
Quartiles				0.80	(0.78, 0.82)	<0.001
Q1 (1738)	1.52	(1.11, 2.08)	0.01			
Q2 (1754)	1.04	(0.75, 1.46)	0.8			
Q3 (1756)	1.17	(0.83, 1.65)	0.4			
Q4 (1749)	1.00					
10th PCTILE				0.80	(0.79, 0.82)	<0.001
Poor	1.74	(1.32, 2.29)	<0.001			
Good	1.00					
**VST-complex (6997)**						
Quartiles				0.80	(0.79, 0.82)	<0.001
Q1 (1740)	1.52	(1.12, 2.06)	0.01			
Q2 (1755)	1.03	(0.74, 1.44)	0.9			
Q3 (1751)	0.92	(0.65, 1.30)	0.6			
Q4 (1751)	1.00					
10th PCTILE				0.81	(0.79, 0.83)	<0.001
Poor	2.17	(1.67, 2.82)	<0.001			
Good	1.00					
**NART Errors (8109)**						
Quartiles				0.80	(0.78, 0.82)	<0.001
Q1 (1782)	0.94	(0.68, 1.28)	0.7			
Q2 (2154)	0.84	(0.63, 1.12)	0.4			
Q3 (1956)	0.81	(0.61, 1.07)	0.1			
Q4 (2027)	1.00					
10th PCTILE				0.80	(0.78, 0.82)	<0.001
Poor	0.94	(0.68, 1.28)	0.7			
Good	1.00					
**Composite score (6151)**						
Quartiles				0.82	(0.80, 0.84)	<0.001
Q1 (1786)	4.01	(2.47, 6.53)	<0.001			
Q2 (1336)	2.24	(1.34, 3.74)	0.002			
Q3 (1370)	1.70	(0.99, 2.91)	0.06			
Q4 (1509)	1.00					
10th PCTILE				0.83	(0.81, 0.85)	<0.001
Poor	3.64	(2.76, 4.80)	<0.001			
Good	1.00					
**Prospec. Memory (8210)**				0.81	(0.80, 0.83)	<0.001
Failure (1531)	2.37	(1.98, 2.84)	<0.001			
Success (6679)	1.00					
**Number of tests**				0.84	(0.82, 0.86)	<0.001
4-8 (N=279)	8.79	(5.77, 13.39)	<0.001			
2-3 (N=1011)	3.82	(2.70, 5.40)	<0.001			
1 (N=1567)	2.04	(2.70, 5.40)	<0.001			
0 (N=3138)	1.00	(1.44, 2.90)	<0.001			

Covariates

Education (the highest level attained) and social class were obtained from the baseline (1993-1997) questionnaire. Education was categorized into three groups: 1) No qualification (not completing school up to the age of 16), 3) Completion of school up to the age of 16 or up to the age of 18; and finally 3) those obtaining an education to graduate level (those who obtained a degree or equivalent) or above. Social class was dichotomized, into 'non manual' and 'manual' class. Age was categorized into 5-year age bands.

Weight was measured to the nearest 0.1 kg (using digital scales, Tanita) and height was measured with a stadiometer (Chasmores, UK) to the nearest 0.1 cm to calculate body mass index (BMI: weight (in kilograms) divided by height (in meters squared)). Lung function was measured by forced expiratory volume in one second (FEV1) and blood pressure was measured by using an Accutorr non-invasive oscillometric blood pressure monitor (Datascope Medical, Huntingdon, United Kingdom) after the participant had been seated for 5 min. Plasma vitamin C levels was estimated using a fluorometric assay. Full details of methods described previously [1].

Self-report of smoking status (current, former or never smoker) and alcohol intake (Units/Week) were obtained from health and lifestyle questionnaire administered at the time of the clinic appointment. Alcohol units were categorized into 3 groups: 0 Units, 1-14 Units, and more than 14 Units. Habitual physical activity was assessed using a four level activity index derived from with two questions referring to activity during the past year [2,3]. For the purposes of the current study, we dichotomized the population into 'physically inactive' (sedentary job and no recreational activity) and 'any physical activity' (any category with activity levels above the latter). Medical history of heart-attack, stroke, cancer, diabetes, hypertension, chronic obstructive pulmonary disease (COPD), depression; and memory and hearing problems, were

established using self-report of a range of conditions from health and lifestyle follow up questionnaire.

Education, social class, physical activity, and smoking were all treated as categorical variables in the analysis, as was co-morbidity (as present or not). Exploratory analyses showed little difference in hazard ratio when BMI was entered as a categorical (as low, normal, overweight and obese groups) or as a continuous variable (data not shown), therefore, BMI was entered in the model as a continuous variable to improve sensitivity of the analysis. The cognitive score was entered as a dichotomized variable based on the description above (poor performance or not).

Creating the Composite Score

For each of the individual cognition tests, a score of '0' or '1' was assigned based on whether the individual was in the 'poor performance' or 'good performance or reference' group for each of the eight cognitive outcome measures individually. The EPIC-COGComp was calculated as a sum of the score based on the performance group for all eight cognition test outcomes (range=0-8). The approximate decile (or obtaining a score of or below 5) for the composite score, was used to define poor performance for general cognition.

REFERENCES

[1] Hayat SA, Luben R, Keevil VL, Moore S, Dalzell N, Bhaniani A, Khawaja AP, Foster P, Brayne C, Wareham NJ, Khaw KT (2014) Cohort profile: A prospective cohort study of objective physical and cognitive capability and visual health in an ageing population of men and women in Norfolk (EPIC-Norfolk 3). *Int J Epidemiol* **43**, 1063–1072.

[2] Wareham NJ, Jakes RW, Rennie KL, Mitchell J, Hennings S, Day NE (2002) Validity and repeatability of the EPIC-Norfolk Physical Activity Questionnaire. *Int J Epidemiol* **31**, 168–174.

[3] Wareham NJ, Jakes RW, Rennie KL, Schuit J, Mitchell J, Hennings S, Day NE (2003) Validity and repeatability of a simple index derived from the short physical activity questionnaire used in the European Prospective Investigation into Cancer and Nutrition (EPIC) study. *Public Health Nutr* **6**, 407–413.
